# IDO1, FAT10, IFI6, and GILT Are Involved in the Antiretroviral Activity of γ-Interferon and IDO1 Restricts Retrovirus Infection by Autophagy Enhancement

**DOI:** 10.3390/cells11142240

**Published:** 2022-07-19

**Authors:** Yoshinao Kubo, Kiyoshi Yasui, Mai Izumida, Hideki Hayashi, Toshifumi Matsuyama

**Affiliations:** 1 Department of Molecular Immunology and Microbiology, Graduate School of Biomedical Sciences, Nagasaki University, Nagasaki 852-8523, Japan; kyasui@nagasaki-u.ac.jp (K.Y.); maimomonga@yahoo.co.jp (M.I.); hhayashi@nagasaki-u.ac.jp (H.H.); toshifumi.matsuyama@gmail.com (T.M.); 2 Department of Clinical Medicine, Institute of Tropical Medicine, Nagasaki University, Nagasaki 852-8523, Japan; 3 Program for Nurturing Global Leaders in Tropical Medicine and Emerging Communicable Diseases, Graduate School of Biomedical Sciences, Nagasaki University, Nagasaki 852-8523, Japan; 4 Medical University Research Administration, Nagasaki University School of Medicine, Nagasaki 852-8523, Japan; 5 Department of Cancer Stem Cell Biology, Institute of Biomedical Sciences, Nagasaki University, Nagasaki 852-8523, Japan

**Keywords:** human immunodeficiency virus type 1, murine leukemia virus, IDO1, FAT10, IFI6, autophagy

## Abstract

Gamma-interferon (γ-IFN) significantly inhibits infection by replication-defective viral vectors derived from the human immunodeficiency virus type 1 (HIV-1) or murine leukemia virus (MLV) but the underlying mechanism remains unclear. Previously we reported that knockdown of γ-IFN-inducible lysosomal thiolreductase (GILT) abrogates the antiviral activity of γ-IFN in TE671 cells but not in HeLa cells, suggesting that other γ-IFN-inducible host factors are involved in its antiviral activity in HeLa cells. We identified cellular factors, the expression of which are induced by γ-IFN in HeLa cells, using a microarray, and analyzed the effects of 11 γ-IFN-induced factors on retroviral vector infection. Our results showed that the exogenous expression of FAT10, IFI6, or IDO1 significantly inhibits both HIV-1- and MLV-based vector infections. The antiviral activity of γ-IFN was decreased in HeLa cells, in which the function of IDO1, IFI6, FAT10, and GILT were simultaneously inhibited. IDO1 is an enzyme that metabolizes an essential amino acid, tryptophan. However, IDO1 did not restrict retroviral vector infection in Atg3-silencing HeLa cells, in which autophagy did not occur. This study found that IDO1, IFI6, FAT10, and GILT are involved in the antiviral activity of γ-IFN, and IDO1 inhibits retroviral infection by inducing autophagy.

## 1. Introduction

Interferons (IFNs) are divided into three types according to their cell surface receptors. Type I IFNs, including α-, β-, ω-, ε-, and κ-IFNs, use heterodimers of IFN α receptor (IFNAR) 1 and 2 as their receptors. In contrast, type II IFNs, including γ-IFN, recognize IFN γ receptor 1 (IFNGR1) as their receptor, while type III IFNs, including λ-IFN, share their receptor protein with interleukin 28 and 29. When host cells detect viruses, they express type I IFNs but not type II and III IFNs. Type I IFNs activate a transcription complex consisting of signal transducer and activator of transcription (STAT) 1 and 2, and IFN regulatory factor 9 (IRF9) proteins and induce the expression of various antiviral factors [1]. To date, many type I IFN-inducible antiviral host factors have been identified [2]. Apolipoprotein B mRNA editing enzyme 3G (APOBEC3G) suppresses the replication of human immunodeficiency virus type 1 (HIV-1) by inducing C-to-T base changes in the reverse-transcribed viral DNA genome [3], and its expression is activated by type I [4] and II IFNs [5]. Other anti-HIV-1 host factors, MX2 [6,7] and interferon induced transmembrane protein 1 (IFITM1) [8], are induced by type I IFNs.

Many cells express type I IFNs upon stimulation by pathogen-specific molecular patterns, but only immune cells, such as T cells and macrophages, express γ-IFN [1,2]. In contrast, many cells express IFNGR1, and γ-IFN inhibits viral replication in many cells. However, the antiviral mechanism of γ-IFN has not been studied in greater detail than that of type I IFNs. Although several antiviral host factors are induced by γ-IFN as well as type I IFN [9], γ-IFN-specific antiviral factors may exist.

**Figure 1:**
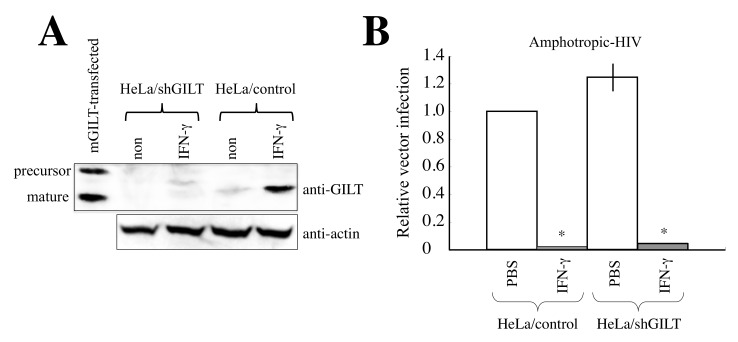
Unknown cellular factors other than GILT inhibit amphotropic MLV-pseudotyped HIV-1-based vector infection in HeLa cells. (**A**) HeLa/control and HeLa/shGILT cells were treated with γ-IFN (0.2 μg/mL). Cell lysates were prepared 3 days after the treatment. The cell lysates were analyzed by Western blotting. (**B**) HeLa/control and HeLa/shGILT cells were treated with γ-IFN for 3 days and were inoculated with amphotropic MLV-pseudotyped HIV-1-based vector. Transduction titers in HeLa/control cells are always set to 1, and relative values ± SD are indicated. Asterisks indicate significant differences compared to PBS-treated cells (see Section 2). This experiment was performed in triplicate.

Previously, we reported that γ-IFN-inducible lysosomal thiolreductase (GILT) functions as an antiretroviral factor [10]. GILT expression is induced by γ-IFN but not by type I IFN. Exogenous expression of GILT suppresses various viral envelope proteins (Envs)-mediated infections [10,11,12]. Mouse embryonic fibroblasts from GILT-deficient mice are more susceptible to virus infection than those from wild-type mice [10,13]. Moreover, GILT silencing by a specific shRNA abrogated the antiviral activity of γ-IFN in TE671 cells, indicating that γ-IFN restricts viral infection by inducing GILT in TE671 cells [10]. However, γ-IFN significantly inhibited amphotropic Env-mediated infection in GILT-silenced HeLa cells (Figure 1). This suggests that unknown host factors other than GILT are involved in the γ-IFN-mediated restriction of viral infection in HeLa cells. We aimed to identify such γ-IFN-inducible host factors in this study.

## 2. Materials and Methods

### 2.1. Plasmids

HIV-1 Gag-Pol-Tat-Rev expression plasmid was provided by Dr. D. Trono [14]. This expression plasmid does not encode any other accessory proteins, Vif, Vpr, Vpu, and Nef. LacZ-encoding HIV-1-based vector genome expression plasmid was obtained from Dr. L. Chang [15]. MLV Gag-Pol expression plasmid was purchased from TaKaRa and does not encode glycosylated Gag protein. Amphotropic MLV Env expression plasmid was constructed in our laboratory [16]. FAT10, IFI6, and GILT shRNA-encoding HIV-1-based vector genome expression plasmids were purchased from Santa Cruz Biotechnology. An expression plasmid of mouse GILT was purchased from Santa Cruz Biotechnology. Renilla luciferase-encoding MLV-based vector genome expression plasmid was constructed in this study.

### 2.2. Cell

Human 293T, HeLa, TE671, and TELCeB6 [17] cells were maintained in our laboratory for a long period. These cells were cultured in Dulbecco’s modified Eagle medium (Wako) with 8% fetal bovine serum and 1% penicillin–streptomycin (Sigma-Aldrich, St. Louis, MI, USA).

### 2.3. cDNA Isolation of γ-IFN-Induced Factors

HeLa cells were treated with γ-IFN (0.2 μg/mL) for 3 days, and total RNA was isolated using TRIzol reagent (Thermo Fisher Scientific, Carlsbad, CA, USA). FAT10, IDO1, tripartite motif-containing protein 22 (TRIM22), retinoic acid receptor responder 3 (RARRES3), IFI27, serpin family G member 1 (SERPING1), apolipoprotein L6 (APOL6), IFI6, and secreted and transmembrane (SECTM) cDNA sequences that contain their whole protein-coding regions were amplified by RT-PCR (TaKaRa) using a total RNA sample from γ-IFN-treated HeLa cells. Guanine nucleotide-binding protein 1 (GBP1) and GBP2 cDNA sequences were amplified by RT-PCR using a total RNA sample from β-IFN-treated 293T cells. The PCR products were cloned into pTargeT mammalian expression plasmid (Promega) or MLV-based vector genome expression plasmid (pMXpuro) [18]. Accession numbers of SERPING1, TRIM22, SECTM, IFI27, IFI6, RARRES3, IDO1, APOL6, FAT10, GBP1, and GBP2 are LC420309, LC420310, LC420311, LC420312, LC420313, LC420314, LC420315, LC420316, LC420317, LC420318, and LC420319, respectively.

### 2.4. Construction of Retroviral Vector

To construct amphotropic MLV-pseudotyped HIV-1-based vector, 293T cells were transfected with the HIV-1 Gag-Pol-Tat-Rev (1 μg), LacZ-encoding HIV-1-based vector genome (1 μg), and amphotropic MLV Env (1 μg) expression plasmids using Fugene transfection reagent (Promega) (5 μL) in a 6 cm-dish. To remove the transfection reagent, culture media were changed to fresh media 24 h after the transfection and continued to be cultured for 24 h. Culture supernatants of the transfected cells were inoculated to target cells. The inoculated cells were stained with X-Gal 2 days after inoculation. The numbers of blue cells were counted in eight randomly selected microscopic fields to estimate transduction titers. This HIV-1-based vector is self-inactivating [15]. Thus, even when cells stably transduced by an HIV-1-based vector are transfected with the HIV-1-based vector construction plasmids, the HIV-1-based vector genome integrated into host cell chromosomal DNA cannot be recovered.

Renilla luciferase (RLuc)-encoding amphotropic MLV-based vector-producing cells were constructed as follows. TELCeB6 cells that stably express MLV Gag-Pol and LucZ-encoding MLV-based vector genome [17] were transfected by the amphotropic MLV Env expression plasmid and were then selected with geneticin (Thermo Fisher Scientific). The geneticin-resistant cell pool (TEL/ampho) was used in the following experiment. To construct RLuc-encoding amphotropic MLV-based vector, 293T cells were transfected with the MLV Gag-Pol, RLuc-encoding MLV-based vector genome, and amphotropic MLV Env expression plasmids. The culture supernatant containing RLuc-encoding amphotropic MLV-based vector was inoculated to the TEL/ampho cell pool. The inoculated TEL/ampho cells were selected with puromycin (Sigma-Aldrich) because the RLuc-encoding MLV-based vector additionally encodes the puromycin-resistant gene. As the RLuc-encoding MLV vector is not self-inactivating, the geneticin- and puromycin-resistant cell pool constitutively produces amphotropic Env-containing, RLuc-encoding MLV-based vector particles. To estimate transduction titers, RLuc activities of cell lysates prepared from inoculated cells were measured by the Renilla luciferase assay system (Promega) 2 days after the inoculation.

Target cells were treated with or without γ-IFN (0.02 or 0.2 μg/mL) for 3 days and washed with PBS to remove γ-IFN. The treated cells were inoculated with the retrovirus vector.

### 2.5. Polymerase Chain Reaction of Unintegrated MLV-Based Vector Genome

Total DNA was isolated from cells 6 h after inoculation with amphotropic MLV-based vector encoding RLuc. PCR (TaKaRa, Otsu, Japan) was performed to detect Renilla luciferase and glyceraldehyde-3-phosphate dehydrogenase (GAPDH) sequences. PCR products were subjected to agarose gel electrophoresis.

### 2.6. Western Immunoblotting

Cells were treated with or without γ-IFN for 3 days, and cell lysates were prepared from the treated cells. Cells were transiently transfected with the mouse GILT expression plasmid or HIV-1-based vector construction plasmids, and cell lysates were prepared from the transfected cells 2 days after the transfection. Cells were treated with or without concanamycin A (CMA) (1 nM) for 1 day, and then cell lysates were prepared from the treated cells.

The protein concentrations of cell lysates were measured using a microBCA assay. Equal protein amounts were subjected to SDS-PAGE. The protein amounts were determined based on a cell lysate with the lowest protein concentration. Proteins separated by SDS-PAGE were transferred onto PVDF membranes. These membranes were then treated with goat anti-GILT (Santa Cruz Biotechnology, Dallas, TX, USA), rabbit anti-LC3, anti-IDO1 (Santa Cruz Biotechnology), or mouse anti-actin (Santa Cruz Biotechnology) antibodies. Then, the membranes were treated with HPR-conjugated anti-mouse IgG antibody, anti-rabbit IgG antibody, or protein G (Bio-Rad, Chicago, IL, USA). Antibody-bound proteins were visualized using ECL reagent (Bio-Rad).

### 2.7. Microarray Analysis

HeLa cells were treated with or without γ-IFN (0.2 μg/mL) for 3 days, and total RNA samples were isolated from the treated cells. The RNA sample preparation for microarray and microarray analysis was performed by Biomatrix Research using the Agilent Whole Human Genome Oligo Microarray ver. 2. Accession number of transcriptional profiling of HeLa cells comparing untreated and IFN-treated HeLa cells is GSE79568, and its Excel file is available in https://nudrive.nagasaki-u.ac.jp/public/qrWIAAwKTsWAeikBcquCn3gP-uya-YV_nx8SKeyVxya3 until 25 July 2025 (password: yoshinao) (accessed on 28 June 2022).

### 2.8. Statistics

Differences between two sets of data were analyzed using Student’s *t*-test and were considered significant at *p* < 0.05.

## 3. Results

### 3.1. Unknown Cellular Factors Other than GILT Are Involved in γ-IFN-Mediated Restriction of HIV-1-Based Vector Infection in HeLa Cells

We previously reported that the GILT-silencing in TE671 cells abrogates the γ-IFN-mediated restriction of infection by an HIV-1-based vector containing VSV-G [10]. To assess whether the same result is observed in HeLa cells, GILT-silenced HeLa cells were constructed. An HIV-1-based vector encoding a scramble control or shRNA against GILT (shGILT) was inoculated into HeLa cells, followed by selection with puromycin. The puromycin-resistant stable cell pool (HeLa/control or HeLa/shGILT) was then used in the subsequent experiments. To confirm the GILT silencing, Western blotting was performed using cell lysates prepared from HeLa/control and HeLa/shGILT cells treated with or without γ-IFN (0.2 μg/mL). A cell lysate prepared from HeLa cells transiently transfected with an expression plasmid of mouse GILT (mGILT) was used as a positive control of Western blotting. Mouse and human GILT proteins are both synthesized as precursors, and then the precursors are digested at their N- and C-terminal domains to the mature proteins. The molecular size of mature mouse GILT was lower than that of mature human GILT as expected (Figure 1A). The precursor was detected in the mGILT-transfected cells but not in the γ-IFN-treated cells because the amount of total GILT protein in the transfected cells was too much to digest completely. The human mature GILT protein was detected in HeLa/control cells 3 days after the γ-IFN treatment but not in HeLa/shGILT, confirming the GILT silencing in HeLa/shGILT cells.

To determine the effect of γ-IFN on the retroviral vector infection, HeLa/control and HeLa/shGILT cells were pretreated with or without γ-IFN (0.2 μg/mL) for 3 days and then were inoculated with amphotropic MLV-pseudotyped HIV-1-based vector. Transduction titers of the HIV-1-based vector were significantly reduced by the γ-IFN treatment of HeLa/control and HeLa/shGILT cells (Figure 1B). This result indicates that cellular factors other than GILT are involved in the γ-IFN-mediated restriction of HIV-1-based vector infection in HeLa cells.

### 3.2. Identification of Antiretroviral Host Factors

To identify cellular factors that are induced by γ-IFN in HeLa cells, we performed a microarray analysis of untreated and γ-IFN-treated HeLa cells. Total RNA was isolated 3 days after the γ-IFN treatment (0.2 μg/mL). The results showed that mRNA levels of already known host restriction factors which inhibit HIV-1 infection, TRIM5α [19], MX2 [6], SAMHD1 [20], SERINC3 [21], SERINC5 [21,22], IFITM2 [8], IFITM3 [8], ApoE [23], and PSGL-1 [24] were not significantly elevated by γ-IFN (Table 1). Although IFITM1 expression was upregulated 5.2 times, mRNA levels of 255 host factors were increased by γ-IFN by more than 10 times. Thus, it was thought that IFITM1 is not a major host factor associated with the γ-IFN-mediated restriction of HIV-1-based vector infection. Previously, we reported that GILT functions as an antiretroviral host factor and that its protein expression is induced by γ-IFN (Figure 1A). However, the GILT mRNA level was not elevated by γ-IFN. This suggests that γ-IFN-mediated GILT protein expression occurs at the post-transcriptional level.

Among γ-IFN-induced cellular factors, major histocompatibility complex components, complements, cytokines, and cellular factors whose functions are already known were excluded. We successfully amplified and cloned cDNA sequences of FAT10, IDO1, TRIM22, RARRES3, IFI27, SERPING1, APOL6, IFI6, and SECTM from γ-IFN-treated HeLa cells by RT-PCR. GBP1 and GBP2 cDNA sequences were amplified from β-IFN-treated 293T cells. Fold induction of these mRNAs by γ-IFN is indicated in Table 2. We analyzed the effects of these 11 host factors on amphotropic MLV-pseudotyped HIV-1-based vector infection.

**Figure 2:**
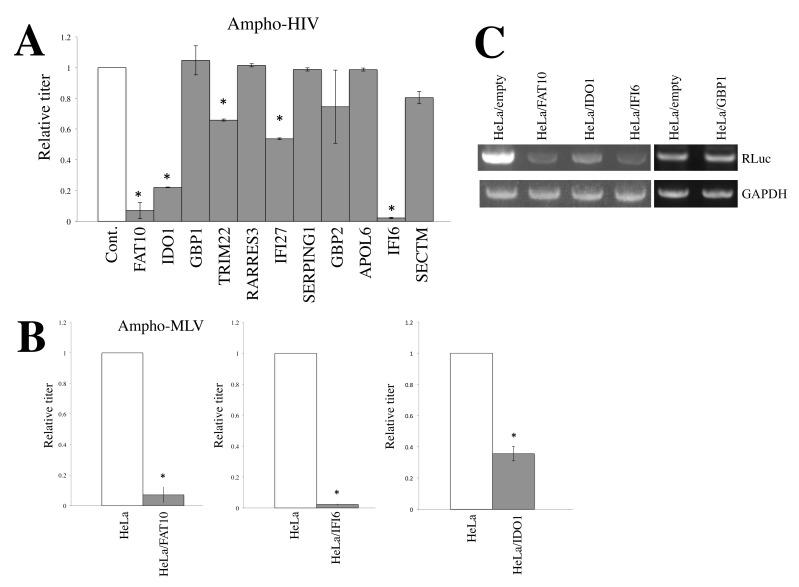
FAT10, IDO1, and IFI6 significantly inhibit amphotropic Env-containing HIV-1- and MLV-based vector infections. (**A**) HeLa cells stably expressing the indicated cellular factors were constructed. These cells were inoculated with amphotropic MLV-pseudotyped HIV-1-based vector. Transduction titers in the control cells are always set to 1, and relative values ± SD are indicated. Asterisks indicate significant differences compared to control cells. This experiment was performed in triplicate. (**B**) FAT10-, IFI6-, or IDO1-expressing HeLa cells were inoculated with amphotropic MLV-based vector, and transduction titers were measured. Asterisks indicate significant differences compared to control HeLa cells. (**C**) Amphotropic MLV-based vector encoding RLuc was inoculated to the indicated cells. Total DNA was isolated 6 h after the inoculation. Rluc sequence was amplified by PCR.

To ensure HeLa cells stably express these host factors, we constructed MLV-based vectors expressing these 11 factors and inoculated them into HeLa cells. The inoculated cells were selected with puromycin because the MLV vector also encodes the puromycin-resistance gene. The stable puromycin-resistant cell pools were used in subsequent experiments. Transduction titers of amphotropic MLV-pseudotyped HIV-1-based vector on the stably transduced cells were measured. The results showed that the transduction titers on the TRIM22- and IFI27-transduced cells were moderately lower than those on the empty vector-transduced cells (Figure 2A). Transduction titers on the FAT10-, IDO1-, and IFI6-expressing HeLa cells (HeLa/FAT10, HeLa/IDO1, and HeLa/IFI6 cells) were much lower than those on the control cells, showing that FAT10, IDO1, and IFI6 significantly inhibit amphotropic MLV-pseudotyped HIV-1-based vector infection. Transduction titers of amphotropic MLV-based vector in HeLa/FAT10, HeLa/IDO1, and HeLa/IFI6 cells were also lower than those in HeLa/empty cells (Figure 2B), which shows that FAT10, IDO1, and IFI6 inhibit MLV-based vector infection as well as HIV-1-based vector infection.

To determine which steps of the HIV-1 life cycle are affected by the three factors, the levels of the reverse transcription products were measured. Total DNA samples were isolated from cells inoculated with an amphotropic MLV-based vector encoding Renilla luciferase (RLuc) 6 h after the inoculation; then, the RLuc sequence was amplified by PCR. The levels of the RLuc PCR product in HeLa/FAT10, HeLa/IDO1, and HeLa/IFI6 cells were lower than those in HeLa/empty and HeLa/GBP1 cells (Figure 2C), showing that these factors inhibit reverse transcription or steps upstream of it in the retroviral life cycle.

### 3.3. Restriction Factors Involved in the Antiretroviral Activity of γ-IFN

To determine whether FAT10, IFI6, and IDO1 are required for the antiretroviral activity of γ-IFN, the expression of FAT10 and IFI6 was silenced by HIV-1-based vectors encoding their specific shRNAs (shFAT10 and shIFI6). Because GILT is γ-IFN-inducible and has antiretroviral activity, the simultaneous inhibition of FAT10, IFI6, and IDO1 functions may have no effect on the γ-IFN antiviral activity in HeLa cells. Thus, GILT expression was also silenced by shGILT [10]. IDO1 (indoleamine 2,3-dioxygenase 1) digests an essential amino acid, tryptophan, and the antiretroviral activity of IDO1 is suppressed by excess tryptophan (see below). Thus, the antiviral function of IDO1 was abrogated by the addition of excess tryptophan (10 μg/mL). All or three of the four factors were simultaneously silenced in HeLa cells.

**Figure 3:**
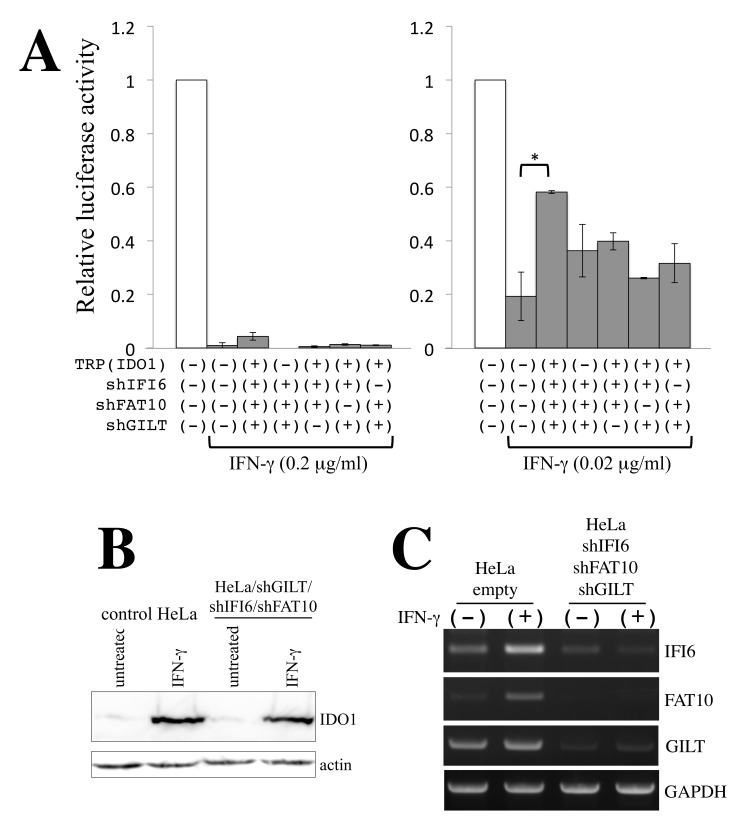
FAT10, IFI6, IDO1, and GILT are all involved in the antiretroviral activity of γ-IFN. (**A**) Amphotropic MLV-based vector encoding Renilla luciferase was inoculated to the indicated cells in the absence or presence of γ-IFN. Luciferase activities in control HeLa cells without γ-IFN are always set to 1, and relative values ± SD are indicated. Asterisks indicate significant differences between the indicated groups. This experiment was performed in triplicate. (**B**) Cell lysates were prepared from indicated cells untreated and treated with γ-IFN and were analyzed by Western blotting. (**C**) Total RNA samples were prepared from the indicated cells in the absence or presence of γ-IFN. Levels of IFI6, FAT10, GILT, and GAPDH were measured by semi-quantitative RT-PCR.

To assess whether the four factors are involved in the antiretroviral function of γ-IFN, HeLa cells expressing shFAT10, shIFI6, and shGILT were pretreated with or without γ-IFN for 3 days and were inoculated with amphotropic MLV-based vector encoding RLuc in the presence of excess tryptophan. When HeLa cells were treated with 0.2 μg/mL γ-IFN, RLuc activity was significantly reduced in all cell pools (Figure 3A, left panel). This suggests that other host factors induced by γ-IFN are involved in the antiviral activity of γ-IFN. However, when HeLa cells were treated with 0.02 μg/mL γ-IFN, simultaneous inhibition of the antiviral functions of all four factors increased RLuc activity (Figure 3A right panel). Simultaneous silencing of three of the four factors did not alter RLuc activity. These results show that FAT10, IFI6, IDO1, and GILT are all involved in the antiretroviral activity of γ-IFN.

Since the function of IDO1 was inhibited by excess tryptophan, the IDO1 protein level in γ-IFN-treated cells was not altered in HeLa cells in which IFI6, FAT10, and GILT were simultaneously silenced (Figure 3B). The levels of FAT10 and IFI6 mRNAs were increased by γ-IFN treatment and were decreased by their specific shRNA (Figure 3C). Consistent with the microarray results (Table 1), the GILT mRNA level was not increased by γ-IFN.

Taken together, these results indicate that all IDO1, IFI6, FAT10, and GILT participate in the antiretroviral activity of γ-IFN. However, unknown host factor(s) are required for full antiviral activity.

### 3.4. IDO1 Inhibits HIV-1-Based Vector Infection through Autophagy Enhanced by Tryptophan Depletion

**Figure 4:**
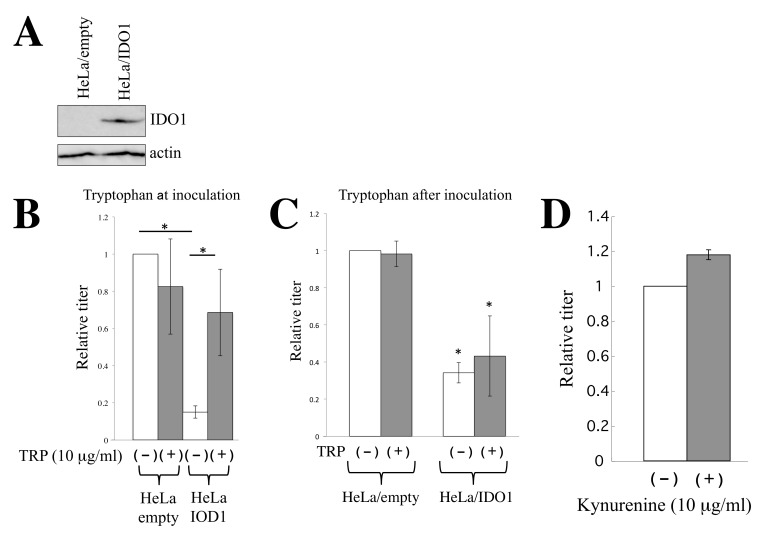
IDO1 inhibits HIV-1 vector infection via tryptophan depletion. (**A**) Cell lysates were prepared from HeLa/empty and HeLa/IDO1 cells. Western blotting of the cell lysates was performed. (**B**) HeLa/empty or HeLa/IDO1 cells were inoculated with amphotropic MLV-pseudotyped HIV-1 vector in the absence or presence of tryptophan (tryptophan at inoculation). Transduction titers in empty MLV vector-transduced HeLa cells without tryptophan are always set to 1, and relative values ± SD are indicated. Asterisks indicate significant differences between the indicated groups. This experiment was performed in triplicate. (**C**) The transduced HeLa cells were inoculated with amphotropic MLV-pseudotyped HIV-1 vector in the absence of tryptophan. The inoculated cells were cultured for 6 days in the absence of tryptophan and were then cultured for 3 days with or without tryptophan (tryptophan after inoculation). The cells were stained with X-Gal. Numbers of blue cells in control HeLa cells without tryptophan are always set to 1, and relative values ± SD are indicated. Asterisks indicate significant differences compared to HeLa/empty cells in the absence of tryptophan. This experiment was performed in triplicate. (**D**) Control HeLa cells were inoculated with amphotropic MLV-pseudotyped HIV-1-based vector in the absence or presence of kynurenine (10 μg/mL). Numbers of blue cells in the absence of kynurenine are always set to 1, and relative values ± SD are indicated. This experiment was performed in triplicate.

We further analyzed the antiviral activity of IDO1 in detail. To examine IDO1 protein expression in HeLa/IDO1 cells, Western blotting was performed using an anti-IDO1 antibody. IDO1 protein was detected in HeLa/IDO1 cells but not in HeLa/empty cells (Figure 4A).

IDO1 encodes indoleamine 2,3-dioxygenase, which metabolizes the essential amino acid tryptophan [25]. To determine whether IDO1 inhibits HIV-1-based vector infection by tryptophan depletion, amphotropic MLV-pseudotyped HIV-1-based vector was inoculated into HeLa/empty or HeLa/IDO1 cells in the presence or absence of excess tryptophan (10 μg/mL). In the HeLa/empty cells, excess tryptophan did not alter the transduction titers of the HIV-1-based vector (Figure 4B). Consistent with the above result, transduction titers in HeLa/IDO1 cells were much lower than those in the HeLa/empty cells. The addition of excess tryptophan significantly elevated the transduction titers in HeLa/IDO1 cells.

There is a possibility that IDO1 inhibits the synthesis of the marker LacZ protein by tryptophan depletion, but not HIV-1-based vector infection. To assess this issue, the cells were inoculated in the absence of tryptophan and were cultured for 6 days. The inoculated cells were additionally cultured for 3 days in the presence of tryptophan and stained with X-Gal (Figure 4C). If IDO1 inhibits LacZ protein synthesis but not vector infection, the number of LacZ-expressing cells would recover. However, the numbers of LacZ-expressing cells were not increased in the presence of excess tryptophan. These results show that IDO1 inhibits HIV-1-based vector infection.

IDO1 generates kynurenine from tryptophan. To know whether kynurenine inhibits HIV-1-based vector infection, target HeLa cells were inoculated with the amphotropic MLV-pseudotyped HIV-1-based vector in the absence or presence of excess kynurenine (10 μg/mL). Transduction titers were not changed by kynurenine (Figure 4D). These results indicate that IDO1 inhibits the HIV-1-based vector infection by the depletion of tryptophan but not by the generation of kynurenine.

**Figure 5:**
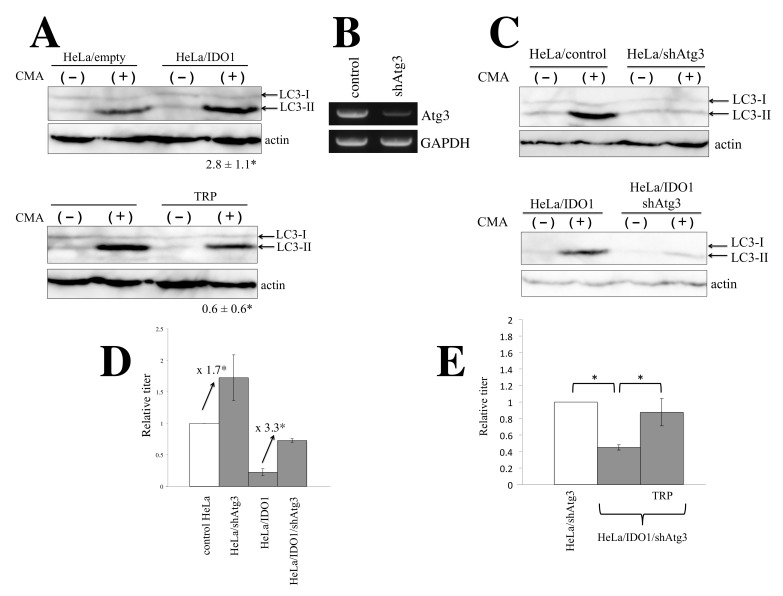
IDO1 inhibits HIV-1-based vector infection through autophagy. (**A**) HeLa/empty or HeLa/IDO1 cells were treated with or without CMA, and cell lysates were prepared. The cell lysates were analyzed by Western blotting using anti-LC3 antibody. Rations of LC3-II to LC3-I were measured. The ratios in CMA-treated control HeLa cells were always set to 1, and relative values ± SD are indicated. Asterisks indicate significant differences compared to CMA-treated HeLa/empty cells. This experiment was performed in triplicate. (**B**) Total RNA samples were prepared from HeLa/control and HeLa/shAtg3 cells. Atg3 mRNA level was measured by semi-quantitative RT-PCR. (**C**) Control and HeLa/IDO1, HeLa/shAtg3, and HeLa/IDO1/shAtg3 cells were treated with or without CMA, and cell lysates were prepared. The cell lysates were analyzed by Western blotting using anti-LC3 antobody. (**D**) Amphotropic MLV-pseudotyped HIV-1-based vector was inoculated to the indicated cells. Transduction titers in control HeLa cells are always set to 1, and relative values ± SD are indicated. Asterisks indicate significant differences between the indicated groups. This experiment was performed in triplicate. (**E**) Amphotropic MLV-pseudotyped HIV-1-based vector was inoculated into indicated cells in the absence of tryptophan. The inoculated cells were cultured for 6 days in the presence of tryptophan and further cultured for 3 days with or without tryptophan. The cells were stained with X-Gal. Numbers of blue cells in HeLa/shAtg3 cells cultured without tryptophan were always set to 1, and relative values ± SD were indicated. Asterisks indicate significant differences between the indicated groups. This experiment was performed in triplicate.

It was reported that IDO1 induces autophagy [26,27]. To confirm this, the levels of LC3-II, an autophagy marker [28], were measured by Western blotting. As LC3-II is degraded in autophagosomes, cells were treated with a lysosome inhibitor, concanamycin A (CMA) [29]. LC3-II levels were significantly increased by CMA treatment of control HeLa cells, suggesting that autophagy constitutively occurs in HeLa cells (Figure 5A upper panel). LC3-II levels were clearly elevated in the IDO1-expressing cells compared with those in the control cells, and excess tryptophan decreased LC3-II levels in HeLa/IDO1 cells. These results show that IDO1 expression facilitates autophagy induction by tryptophan depletion.

There are several lines of evidence suggesting that autophagy inhibits HIV-1 infection [30,31,32]. To examine whether IDO1 inhibits HIV-1-based vector infection by enhancing autophagy, HeLa/empty and HeLa/IDO1 cells were further transduced by an HIV-1-based vector encoding an shRNA against Atg3 (shAtg3), which is required for autophagy induction [33]. HeLa/IDO1 cells were already resistant to puromycin. When puromycin-sensitive HeLa cells were inoculated with VSV-pseudotyped HIV-1-based vector encoding shAtg3 and selected with puromycin, almost all cells survived. Thus, many of the HeLa/IDO1 cells inoculated with the shAtg3-encoding HIV-1-based vector (HeLa/IDO1/shAtg3 cells) would express shAtg3. Atg3 mRNA level was indeed decreased in HeLa/IDO1/shAtg3 cells compared with the control cells (Figure 5B). LC3-II levels in HeLa/shAtg3 and HeLa/IDO1/shAtg3 cells were much lower than those in control HeLa and HeLa/IDO1 cells (Figure 5C), indicating that Atg3 silencing inhibits autophagy induction. Transduction titers of amphotropic MLV-pseudotyped HIV-1-based vector were elevated 1.7 and 3.3 times by the Atg3 silencing in control and HeLa/IDO1 cells, respectively (Figure 5D). Taking these findings together, it is suggested that IDO1 inhibits the HIV-1 vector infection through autophagy facilitated by tryptophan depletion.

However, transduction titers in HeLa/IDO1/shAtg3 cells were lower than those in HeLa/shAtg3 cells, showing that IDO1 expression inhibited HIV-1-based vector infection in the Atg3-silenced cells. To examine whether IDO1 inhibits LacZ reporter protein synthesis by tryptophan depletion in Atg3-silenced cells, HeLa/IDO1/shAtg3 cells were inoculated with amphotropic MLV-pseudotyped HIV-1-based vector in the absence of excess tryptophan. The inoculated cells were cultured for 6 days and then cultured for 3 days in the presence of excess tryptophan. The numbers of LacZ-expressing cells were increased by excess tryptophan (Figure 5E). This result indicates that IDO1 expression inhibits LacZ protein synthesis, but not HIV-1-based vector infection, in the Atg3-silenced cells, and thus the antiviral activity of IDO1 requires autophagy.

**Figure 6:**
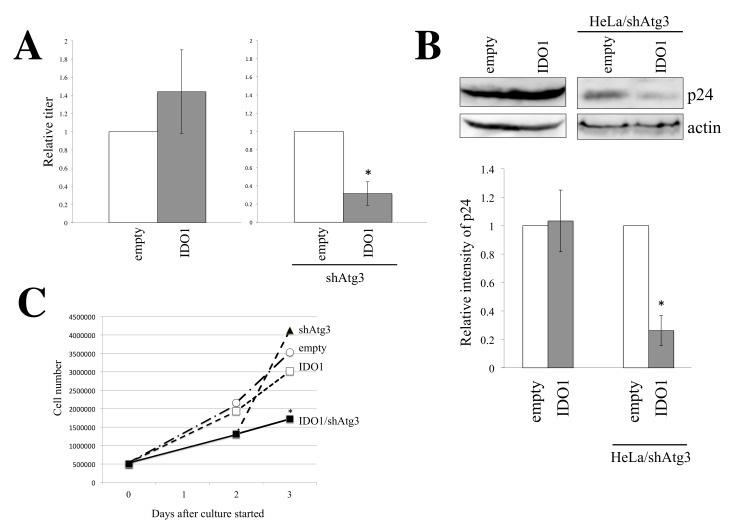
IDO1 does not affect HIV-1 particle production. (**A**) Indicated cells were transfected with the amphotropic MLV-pseudotyped HIV-1-based vector construction plasmids. Culture supernatants of the transfected cells were inoculated to TE671 cells. Transduction titers from control HeLa cells are always set to 1, and relative values ± SD are indicated. This experiment was performed in triplicate. Asterisks indicate significant differences compared to HeLa/shAtg3 cells. (**B**) Cell lysates were prepared from the transfected cells and were analyzed by Western blotting using anti-HIV-1 p24 antibody. Band intensities of p24 protein in control HeLa cells were always set to 1, and relative values ± SD are indicated. This experiment was performed in triplicate. Asterisks indicate significant differences compared to HeLa/shAtg3 cells. (**C**) Indicated cells were cultured in media containing 4% FBS, and numbers of cells were counted every day. This experiment was performed in triplicate. SD is not shown because error bars make the graph busy. Asterisks indicate significant differences compared to HeLa/empty cells at the same time.

To examine the effect of IDO1 expression on HIV-1 particle production, HeLa/empty, HeLa/IDO1, HeLa/shAtg3, and HeLa/IDO1/shAtg3 cells were transfected by amphotropic MLV-pseudotyped HIV-1-based vector construction plasmids. Culture supernatants were inoculated to TE671 cells to measure transduction titers. IDO1 expression reduced transduction titers (Figure 6A) and p24 levels (Figure 6B) in the Atg3-silenced cells but not in control HeLa cells. These results show that in the Atg3-silenced cells, IDO1 expression inhibits the synthesis of HIV-1 Gag and LacZ proteins.

The above result suggests that IDO1 expression in Atg3-silenced cells inhibits total protein synthesis due to tryptophan depletion. To assess this speculation, cell growth was analyzed. As expected, numbers of HeLa/IDO1/shAtg3 cells were lower than those of other cells in the 4% FBS-containing medium (Figure 6C). Taken together, it was found that IDO1 expression in Atg3-silenced cells inhibits cell growth through the inhibition of total protein synthesis.

## 4. Discussion

We identified novel γ-IFN-induced antiretroviral host factors, FAT10, IFI6, and IDO1, in this study. Exogenous expression of these factors significantly suppressed retroviral vector infection. Simultaneous knockdown of all of these factors diminished the antiretroviral activity of γ-IFN.

To the best of our knowledge, this is the first report showing that FAT10 functions as a host factor acting against retroviruses. Moreover, no reports have shown that FAT10 inhibits not only retroviruses but also other viruses. The effects of type I IFN-induced cellular factors on HIV-1 infection were comprehensively analyzed [34]. Because FAT10 expression is induced by type II IFN, FAT10 was not analyzed in that previous study. In contrast to our results, it was reported that FAT10 inhibits RIG-I-induced antiviral activity [35] and is required for influenza A virus replication by inhibiting type I IFN signaling [36]. Because retroviral vector infection in HeLa cells does not induce type I IFN, this function of FAT10 is not associated with its antiretroviral activity. It is known that FAT10 is a ubiquitin-like modifier of proteins and induces the proteasomal degradation of target proteins [25]. Thus, FAT10 may inhibit retroviral infection by degrading viral proteins or cellular proteins required for such infection. Further study is required to clarify the mechanism behind the antiretroviral activity of FAT10.

We found that IFI6 restricts retroviral infection. It was previously reported that IFI6 inhibits the replication of flaviviruses [34,37,38,39,40]. However, a report has also described that IFI6 actually promotes hepatitis C virus replication [41]. In contrast to our result, Schoggins et al. showed that IFI6 had no effect on HIV-1 infection in Jurkat T cells [34]. The antiviral activity of IFI6 may thus depend on the cell line used, but further study is required to clarify this.

It has been already reported that IDO1 restricts many viral infections [26,42,43,44]. However, the mechanism of IDO1 antiviral activity has not been understood yet. Because IDO1 generates kynurenine from tryptophan, the cellular concentrations of tryptophan and kynurenine are decreased and increased by IDO1 expression, respectively. It has been reported that kynurenine induces several neurological and immunological alterations [45,46]. However, excess tryptophan overcame the antiviral activity of IDO1. This result shows that the depletion of tryptophan but not the generation of kynurenine inhibits vector infection because excess tryptophan does not attenuate the IDO-mediated production of kynurenine and other metabolites. Indeed, excess kynurenine did not affect the retroviral vector infection. The biological changes induced by kynurenine and other metabolites generated by IDO1 are not involved in the antiviral activity of IDO1. However, the biological events induced by the tryptophan depletion other than autophagy may also participate in antiviral activity.

**Figure 7:**
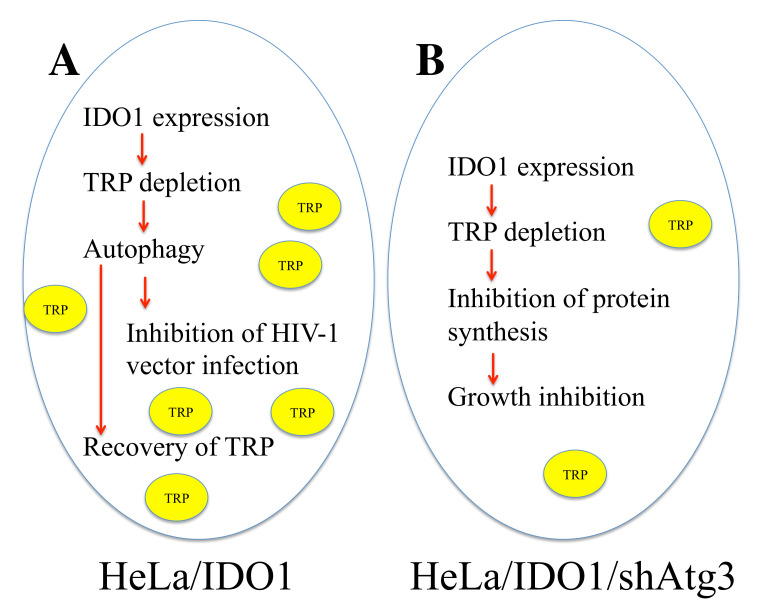
Mechanism of IDO1-mediated inhibition of HIV-1 infection. (**A**) In IDO1-expressing HeLa cells, tryptophan is digested, and cellular tryptophan level is decreased. As a result, autophagy is enhanced. HIV-1 vector infection is inhibited by activated autophagy. Entered HIV-1 particles may be degraded in autophagosomes. On the other hand, proteins and organelle are digested by autophagy, and tryptophan level is recovered. Thus, protein synthesis and cell growth are not suppressed. (**B**) In HeLa/IDO1/shAtg3 cells, IDO1 digests tryptophan, and cellular tryptophan level is reduced. However, autophagy is not enhanced by Atg3 silencing, and tryptophan level is not recovered. Thus, protein synthesis and cell growth are suppressed.

This study found that the IDO1-mediated tryptophan depletion inhibits retrovirus infection via enhanced autophagy (Figure 7). The enhanced autophagy degrades organelles and proteins, recovering the cellular tryptophan level. Thus, total protein synthesis is not inhibited. In Atg3-silenced cells, IDO1 digests tryptophan, but autophagy is not enhanced. Therefore, the cellular tryptophan level is not recovered. The synthesis of total proteins, including viral, marker, and cellular proteins, is inhibited, and cell growth is suppressed. When the same amounts of cell lysates were subjected to SDS-PAGE, HIV-1 p24 levels were decreased in HeLa/IDO1/Atg3 cells but not in HeLa/IDO1 cells. The aberrant expression of HIV-1 Gag protein in transiently transfected cells may be more significantly affected by the tryptophan depletion than the expression of cellular proteins. Autophagy may digest invading retroviral particles together with cellular organelles and proteins and thus inhibit retrovirus infection.

It has already been reported that IDO1 expression in target cells suppresses HIV-2 infection, but not HIV-1 infection, and IDO1 inhibits the expression of HIV-1 proteins [26]. The authors used a single-cycle infection assay by GFP-expressing HIV-1 or HIV-2 vector and analyzed GFP-positive cells to measure viral titers. Although IDO1 inhibits viral and GFP protein expressions from the HIV-2 vector by tryptophan depletion, GFP maker expression from the HIV-1 vector is not decreased by IDO1. If it is true that the synthesis of the HIV-2 proteins is inhibited by the IDO1-mediated tryptophan depletion, the synthesis of GFP and cellular proteins would also be inhibited. However, we showed here that IDO1 expression did not affect the expression of HIV-1 and LacZ maker proteins. The mechanism by which the tryptophan depletion selectively inhibits the HIV-2 protein synthesis has not been clarified in that study [26]. In our study, IDO1 activated autophagy by tryptophan depletion. It is widely accepted that autophagy degrades many infectious microbes and inhibits their infection [30,31,32]. The cellular level of tryptophan may be partially compensated by the digestion of organelles and proteins in autophagy. Thus, tryptophan levels in culture media may affect the antiviral activity of IDO1. Alternatively, one of the HIV-1 accessory proteins, but not HIV-2 accessory proteins, may attenuate the IDO-mediated antiviral activity because the replication-defective HIV-1-based vector used in this study does not encode any HIV-1 accessory proteins.

The accessory proteins of HIV-1 inhibit the antiviral activities of host restriction factors by inducing their degradation [47]. However, GILT expression was inhibited, and GILT silencing enhanced the replication of HIV-1 encoding all accessory proteins [10]. Thus, the antiviral activity of GILT is not inhibited by the HIV-1 proteins. We previously reported that γ-IFN does not restrict HIV-1 proliferation through the suppression of γ-IFN-mediated signaling by the HIV-1 Env protein, and the treatment of HIV-1-infected cells with γ-IFN does not induce GILT, IFI6, and FAT10 [10]. Therefore, HIV-1 may not need the accessory proteins to inhibit these host antiviral factors.

The GILT silencing abrogated the antiviral activity of γ-IFN in TE671 cells but not in HeLa cells, although GILT protein is induced by γ-IFN treatment of HeLa cells. It has been shown that the cell type specificity of the γ-IFN antiviral activity is correlated with the induction of IDO1 [43]. However, this study found that γ-IFN-induced host factors other than IDO1, FAT10, IFI6, and GILT are required for the full antiviral activity of γ-IFN. It is unclear whether such host factors are not induced by γ-IFN or do not function in TE671 cells.

## 5. Conclusions

We found that γ-IFN restricts retroviral vector infection by inducing FAT10, IFI6, IDO1, and GILT in HeLa cells. IDO1 inhibits retroviral vector infection through autophagy activation mediated by tryptophan depletion. Other unknown antiretroviral factors induced by γ-IFN are waiting to be identified.

## Figures and Tables

**Table 1:** Induction of already known restriction factors by γ-IFN.

Host Factors	Untreated	γ-IFN	Fold Induction
TRIM5a	39	33	0.8
MX2	115	97	0.8
SAMHD1	251	297	1.1
SERINC3	4059	2691	0.7
SERINC5	94	115	1.2
IFITM1	3553	18,345	5.2
IFITM2	38,265	46,579	1.2
IFITM3	22,554	23,043	1
ApoE	2062	3193	1.5
PSGL-1	104	73	0.7
GILT	22147	17304	0.8

**Table 2:** Fold induction of γ-IFN-induced factors by γ-IFN.

Host Factors	Untreated	γ-IFN	Fold Induction
FAT10	9	7862	898.3
IDO1	10	4038	413.3
GBP1	9	2481	280.8
TRIM22	11	829	76.3
IFI27	111	6136	55.2
SERPING1	57	2882	50.2
GBP2	7	323	44.8
RARRES3	761	10,985	14.3
APOL6	1086	14,886	13.7
IFI6	215	2860	13.3
SECTM	2645	11,344	4.3

## Data Availability

The result of the microarray analysis of HeLa cells treated with or without γ-IFN is available in https://nudrive.nagasaki-u.ac.jp/public/qrWIAAwKTsWAeikBcquCn3gP-uya-YV_nx8SKeyVxya3 until 25 July 2025 (password: yoshinao) (accessed on 28 June 2022).
